# Survival of Ceramic Veneers: Impact of Dentin Exposure and Tooth Vitality After 1 to 15 Years of Follow‐Up

**DOI:** 10.1111/jerd.70016

**Published:** 2025-08-03

**Authors:** Olivier Etienne, Caroline Jiamiao Wang, Rim Bourgi, Dominique Watzki, Tatiana Roman

**Affiliations:** ^1^ DDS, Department of Prosthodontics, Robert Frank Faculty of Dental Surgery University of Strasbourg Strasbourg France; ^2^ Inserm UMR 1121, “Biomaterials & Bioengineering”, CRBS Strasbourg France; ^3^ CNRS UMR 7357, ICube Illkirch‐Graffenstaden France; ^4^ DDS, Department of Restorative Dentistry, School of Dentistry Saint‐Joseph University Beirut Lebanon; ^5^ Department of Restorative Sciences, Faculty of Dentistry Beirut Arab University Beirut Lebanon; ^6^ Ceramist Illkirch‐Graffenstaden France

**Keywords:** ceramic veneers, dentin exposure, dual‐cure resin cements, enamel preservation, Kaplan–Meier survival, retrospective study

## Abstract

**Objective:**

This retrospective clinical study aimed to evaluate the survival rates of ceramic laminate veneers in relation to dentin exposure, endodontic treatment, preparation design, and the type of dual‐cure resin cement used.

**Materials and Methods:**

A total of 672 veneers were placed in 189 patients and followed for 1 to 15 years. Veneer supporting teeth were categorized after etching based on the degree of dentin exposure into three groups: GA1 (enamel only), GA2 (< 30% dentin), and GA3 (> 30% dentin). Additional variables included endodontic treatment, preparation design, and the resin cement system used. Kaplan–Meier survival analysis, Log‐Rank tests (*α* = 0.05), and Odds Ratios (ORs) were computed. All procedures were performed by a single clinician, veneers were fabricated by a single ceramist, and the study was performed by a different, single examiner.

**Results:**

The cumulative 15‐year estimated survival rate was 96%, mean survival 5.98 years. Veneers in the GA1 group showed an estimated survival rate of 96.7%, compared to 95.3% in GA2 and 93.9% in GA3. A statistically significant difference was found between GA1 and GA3 (*p* = 0.033). Endodontically treated teeth and those with dentin exposure exhibited higher risks of failure (OR = 1.68 and 3.47, respectively). Endodontic treatment, preparation design, and resin cement type did not significantly affect survival (*p* > 0.05).

**Conclusions:**

The extent of dentin exposure significantly impacts the survival of bonded ceramic veneers after 1 to 15 years of follow‐up. Preservation of enamel is critical for optimizing outcomes.

**Clinical Significance:**

These findings suggest that meticulous patient selection, preparation techniques, optimal bonding protocols, and bonding exclusively to enamel, whenever possible, are essential for ensuring the clinical success of CLV bonded with dual‐cured resin.

## 1 Introduction

Ceramic laminate veneers (CLVs) have become a cornerstone of minimally invasive esthetic dentistry, offering a long‐term and predictable conservative solution for the restoration of anterior teeth affected by discoloration, malformation, trauma, wear, or misalignment [1, 2, 3, 4, 5, 6, 7, 8, 9, 10]. The preservation of enamel during tooth preparation for CLVs has emerged as a critical factor for optimizing the adhesive bonding, minimizing the failure risk, and extending the restoration longevity.

In fact, enamel provides an ideal substrate due to its high inorganic content, low water permeability, and stable microstructure, facilitating durable micromechanical and chemical bonding with resin cements [11, 12]. In contrast, dentin poses a complex adhesive challenge, being more hydrated, organic, and less mineralized than enamel. In addition, the collagen matrix and tubular structure of dentin compromise the stability and strength of the resin‐dentin interface, particularly in clinical conditions, where contamination and polymerization limitations may exist [6, 11, 12, 13, 14].

Studies evaluating enamel thickness after preparation confirm that most of the anterior teeth provide sufficient enamel for veneer bonding when the depth reduction is limited to 0.3–0.5 mm at the midfacial third and 0.7–1.0 mm at the incisal third of the tooth [15, 16, 17]. In this context, conservative preparations have been made possible with the Aesthetic Pre‐evaluative Temporary (APT) and mock‐up techniques, enabling controlled depth reduction using mock‐ups and depth‐limiting burs [1, 18]. However, the cervical region typically has only 0.3–0.5 mm of enamel thickness. This makes dentin exposure more likely in the subgingival margins when correcting significant dyschromia or alignment issues, or in cases involving retreatment after a previous invasive restoration [8, 16, 17, 19, 20].

Several clinical studies and systematic reviews have indicated that dentin exposure has a detrimental effect on the survival of CLV, particularly when more than 30% of the bonding surface is situated within dentin [6, 10, 21, 22, 23, 24]. A 12‐year retrospective study found a 99% survival rate for veneers bonded entirely to enamel, compared to 94% for those bonded to mixed (enamel and dentin) substrates [25]. Similarly, it has been reported that veneers bonded to enamel or minimally exposed dentin (< 30%) performed significantly better than those bonded to severely exposed dentin (> 30%) over a 2‐year follow‐up [24]. Furthermore, a comprehensive systematic review concluded that CLV bonded to enamel demonstrated consistently higher long‐term survival and fewer complications than those bonded to dentin [21].

When dentinal exposure is important and cannot be avoided, the Immediate Dentin Sealing (IDS) protocol has been proposed [26]. Initially described as a means of covering the exposed dentin after crown preparation [26], it has also been suggested for CLV preparations. This approach demonstrated a higher survival rate in teeth with more than 50% of dentin exposure when IDS was used (96.4% vs. 81.8%) in an 11‐year prospective clinical trial [10].

Despite the substantial body of evidence supporting the role of enamel preservation and the influence of dentin exposure on CLV performance, few studies have attempted to quantify the clinical outcomes using survival curves stratified by dentin exposure levels [6, 10, 25]. The present study aims to address this gap by conducting a retrospective analysis of the CLV placed over a 15‐year period, with veneers categorized by the extent of dentin exposure: GA1 (0% dentin), GA2 (< 30% dentin), and GA3 (> 30% dentin).

Thus, the primary research hypothesis tested in this study is that dentin exposure affects veneer survival.

In addition, the preparation design, particularly in terms of incisal coverage, also influences veneer survival [27, 28]. Indeed, butt‐margin, window, or incisal overlap preparation design can impact stress distribution, bonding area, and veneer fit [28]. Various classifications have been proposed based on the aggressivity of the preparation, ranging from no‐prep and minimal‐prep designs to conventional and extended designs [29]. In a retrospective study, veneers with incisal overlap demonstrated similar survival to non‐overlapping designs; however, designs with extensive dentin exposure (> 50%) were significantly more prone to biological and technical complications [6].

Another debated factor regarding veneer survival is the endodontic status of the tooth [10, 23, 27, 30, 31]. While endodontically treated teeth may exhibit reduced biomechanical resilience and increased brittleness [31], a consensus has not yet been reached on whether veneer performance is affected by prior root canal treatment. Some reports suggest a higher risk of failure in non‐vital teeth due to an altered bonding behavior and increased flexural stresses [27, 31]. However, other studies have demonstrated comparable clinical longevity between vital and non‐vital teeth restored with CLV [10, 23, 30].

Furthermore, resin cement type and polymerization mode also affect veneer outcomes. Dual‐cure resin cements offer an improved polymerization for thicker or less translucent restorations, but may exhibit lower color stability over time, particularly when aromatic amines are involved in the redox initiation system [32, 33]. On the other hand, light‐cured cements provide excellent esthetic control, but their limited polymerization depth may compromise bonding in thicker restorations or less translucent ceramics [34, 35]. The development of amine‐free dual‐cure systems has sought to overcome these limitations, offering comparable esthetic performance and superior bond strength [13, 35]. At the same time, clinical studies have reported important survival and success rates for veneers bonded with dual‐cured resin cements [25, 36].

These additional variables, including endodontic treatment, preparation design, and resin cement type, were evaluated for their effect on the survival of CLV. Thus, the secondary research hypotheses state that neither the endodontic treatment, the preparation design, nor the resin cement system influences clinical outcomes.

## 2 Materials and Methods

### 2.1 Study Design

A single‐centered retrospective clinical study was conducted on all the patients who had undergone treatment for CLV between July 2001 and January 2023. The study was approved by the Ethics Committee of the Faculty of Dental Surgery Robert FRANK, Strasbourg/France (file number CE‐2023‐10). All patients have provided written consent for the treatment and the use of their photographs, radiological examinations, and master casts.

All clinical procedures, including preparation, bonding, and follow‐up, were performed by a single practitioner (OE), while the veneers were fabricated by a single ceramist (DW). Data for the analysis were extracted from the medical records, photographs, and radiographs and were independently evaluated by a single examiner blind to the studied factors.

### 2.2 Inclusion and Exclusion Criteria

All the veneers that had been placed on the incisors and canines of the maxilla and mandibula have been included. Patients were required to be at least 18 years old by the time of the veneer restoration, and not at high risk of developing caries. Patients with a history of bruxism were not excluded from the study; however, they had been required to utilize occlusal protection in the form of a rigid occlusal splint worn at night [37]. For patients with a history of nail biting (onychophagia), cessation of this habit was a prerequisite for the indication of veneer restorations. Teeth that had undergone endodontic treatment were not excluded from the study. Additionally, patients exhibiting greater tooth structure loss, frequently resulting from wear or extensive restorations have also been included.

The following exclusion parameters were established: (1) veneers featuring 360° contours, chips, or partial veneers with chamfer preparations, owing to their distinct mechanical properties as opposed to butt‐margin or incisal‐overlap designs, (2) patients exhibiting persistent onychophagia, and (3) cases with insufficient documentation, specifically those lacking photographic records.

#### 2.2.1 Data Collection

Patient data for the study (gender, age, location and number of veneers, the dates of veneer placement, reported complications and follow‐up appointments) were retrieved from the patient files (Julie Solutions, Paris, France) for all patients who had a veneer placed between July 2001 and January 2023. Radiographic images were examined to determine whether endodontic treatment had been performed prior to veneer treatment. Photographs taken before, during, and after the placement of veneer restorations allowed for the evaluation of various parameters, including indication for veneer, number and location of the restorations (confirmed with medical records), preparation design, nature of the substrate (differentiating dentin from enamel), and any reported complications. Clinical and photographic records were anonymized using Excel software (Microsoft Corporation, Redmond, USA) to ensure confidentiality.

#### 2.2.2 Teeth Preparation Protocols

**FIGURE 1:**
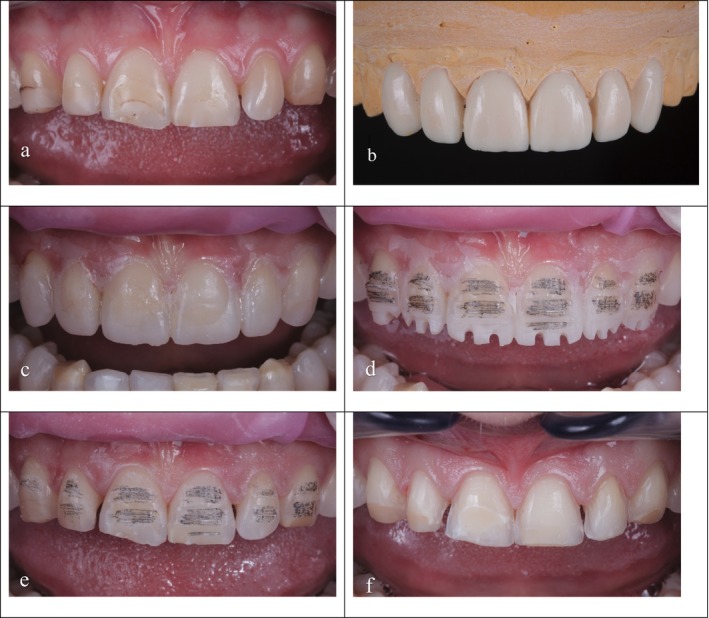
All preparations were conducted following the esthetic pre‐evaluate temporary (APT) technique. Buccal view of the initial clinical situation (a). Wax‐up on the plaster model of anterior teeth (b). Acrylic resin mock‐up in place (c). First stage of preparation carried out with a calibrated depth bur (d). Preparations after mock‐up removal and pencil depth marking (e). Finished butt‐margin preparations (f).

Teeth were prepared with a rigorous and meticulous mock‐up driven protocol in accordance with Aesthetic Pre‐evaluate Temporary (APT) technique (Figure 1) [1].

#### 2.2.3 Bonding Protocol

**FIGURE 2:**
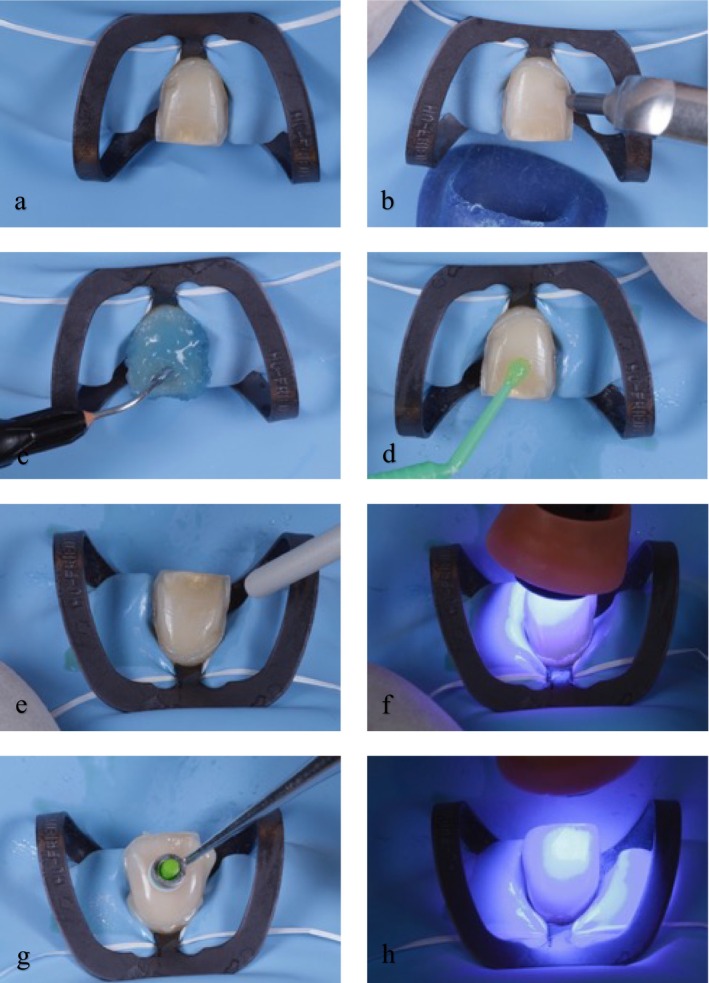
Different stages in the surface treatment of dental substrates. Tooth isolation (a), enamel sandblasting (27 μm alumina oxide, Micro‐etcher II, Danville, USA) (b), etching (c), adhesive application (d), adhesive drying and polymerization (800 mW/cm^2^ for 20s) (e, f). Resin cement application and veneer seating with final polymerization (1200 mW/cm^2^ for 20s on each side) (g, h).

In this study, the bonding protocol was carried out using a total‐etch adhesive approach with a dual‐cured resin cement (Figure 2) [13]. From 2001 to 2015, ExciTE F DSC adhesive (Ivoclar Vivadent, Schaan, Liechtenstein) was used in combination with Variolink II resin cement (VL, Ivoclar Vivadent, Schaan, Liechtenstein). After 2015, the bonding procedure was performed with G‐Premio BOND universal adhesive (GC, Tokyo, Japan) and G‐CEM LinkForce resin cement (GC, Tokyo, Japan). The ceramic material used for all the veneers was the lithium disilicate glass–ceramic (IPS e.max Press; Ivoclar Vivadent, Schaan, Liechtenstein) [38]. The veneer intaglio was etched with a 4.5% hydrofluoric acid gel (IPS e.max etching gel; Ivoclar Vivadent, Schaan, Liechtenstein) for 20 s, followed by rinsing and drying, after which silane was applied for 1 min. Between 2001 and 2015, Monobond Plus (Ivoclar Vivadent, Schaan, Liechtenstein) was used for silanization, and from 2015 to 2023, G‐Multi PRIMER (GC, Tokyo, Japan) has been utilized.

#### 2.2.4 Evaluation Methods

The restorations were carefully assessed during regular follow‐up sessions every 6 months. Patients were also questioned about any post‐operative complications and instructed to report any issues promptly. As a result, complications and failures that occurred prior to each evaluation were documented in the patients' files and included in the final analysis.

In this investigation, clinical survival was defined as the presence of the restoration in situ. Failure events were categorized as circumstances resulting in the loss of the supporting dental structure or necessitating either veneer rebonding or replacement. These failures were classified as either mechanical (fracture, chipping, debonding), biological (post‐operative sensitivities, secondary caries), or esthetical. Complications that did not necessitate veneer rebonding or replacement have also been noted (e.g., minor chipping) but not recorded as failures.

In this study, tooth surfaces were evaluated through photographs using a Canon EOS 7D digital camera with a Tamron SP 90 mm f/2.8 Di VC USD macro lens and twin flashes. The standardized orthogonal buccal views were taken after the phosphoric acid etching stage. For preparations involving an incisal overlap, only the buccal surface was considered.

**FIGURE 3:**
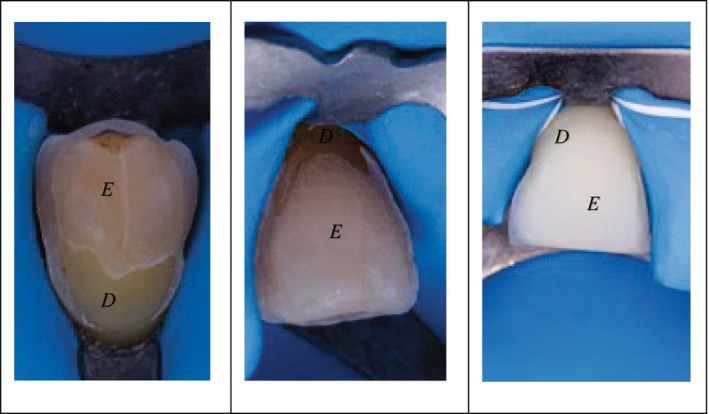
Dental substrate after etching, rinsing, and drying. Post‐etching photographs make it possible to differentiate between the different dental tissues: enamel (E)‐dentin (D).

In the post‐etching photographs, after rinsing and drying, enamel and dentin could be clearly differentiated (Figure 3). Enamel typically remains dry with a matte, chalky appearance, while dentin appears glossy due to intrinsic moisture. Additionally, the enamel is more translucent, while the dentin has a higher chroma, greater opacity, and a smoother surface. Lastly, the striations left by diamond burs are more noticeable on enamel, presenting as whitish streaks. The intact interproximal enamel surrounding the prepared area served as a reference point for the examiner in distinguishing between enamel and dentin.

#### 2.2.5 Assessment of Dentin Exposure

An image processing software, ImageJ (National Institutes of Health, Bethesda, MD, USA) [39], was used by a single‐blind examiner (CW) to visually analyze the photography for each tooth and to numerically quantify the proportion of dentin exposure.

**FIGURE 4:**
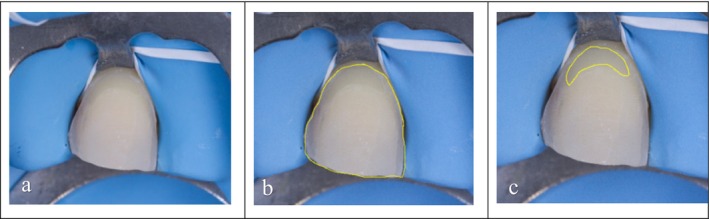
Protocol for evaluating the dental tissues using ImageJ. Importing the post‐etching photograph of the tooth into ImageJ (a). First, the total surface area of the buccal preparation was visually selected and calculated (b). Next, the range of the exposed dentin was selected and measured as a proportion of the total surface (c).

The percentage of exposed dentin area relative to the total vestibular area of the preparation was calculated using the “freehand tool” (Figure 4). The teeth were classified into 3 groups according to the percentage of the exposed dentin: GA1 (enamel‐only, no dentinal exposure), GA2 (< 30% dentin), and GA3 (> 30% dentin).

### 2.3 Statistical Analysis of Survival Rate

Survival analysis was conducted using IBM SPSS Statistics software (version 29.0.0.0 (241); IBM Corporation, Armonk, NY, USA) with the Kaplan–Meier survival test and Log‐Rank (Mantel‐Cox) pairwise comparison, based on parameters such as dentin exposure, endodontic treatment, preparation design, and bonding system used. Censoring events wereloss to follow‐up or the end of the study [40]. The odds ratio for failure, without failure or without any complications, was calculated for tooth vitality and dentinal exposure. Statistical significance was set at 0.05 for all tests.

## 3 Results

Of the 890 veneers initially selected, 650 have been included in the study, for a total of 189 patients (137 women and 52 men). Two hundred and forty veneers have not been included because post‐etching photographs were lacking or uninterpretable. Patients lost to follow‐up were labeled as censuring events, with last known status taken into account. The observation period was 5.98 years (ranging from 1 to 180 months). The mean age of the individuals included in the study was 47 years (ranging from 18 to 90 years at the time of the veneer restoration). Most of the veneers were prepared using the butt‐margin preparation design on vital teeth (VT) of the maxilla. Fifteen percent of the veneers were prepared on mandibular teeth. Overall, 382 teeth showed dentinal exposure (GA2 and GA3 groups). Only 38 teeth were endodontically treated (NVT). The survival, failure, and survival without complications, respective of dentinal exposure, endodontic treatment, preparation design, and bonding system are available in Table 1.

**TABLE 1:** Survival, failure, and survival without complications respective of dentinal exposure, endodontic treatment, preparation design, and bonding system for the included veneer‐supporting teeth.

Ceramic veneers	*N*	Percentage	Failures	Observed survival	Complications, comprising failures	Observed survival without complication
Dentinal exposure						
GA1	290	43.1%	2	99.3%	9	96.8%
GA2	306	45.5%	5	98.4%	12	96.1%
GA3	76	11.3%	4	84.7%	8	89.5%
Endodontic treatment						
With endodontic treatment (NVT)	38	6%	1	97.4%	2	94.7%
Without endodontic treatment (VT)	634	94%	10	98.4%	27	95.7%
Preparation design						
Butt‐margin	650	97%	11	98.3%	29	95.5%
With incisal overlap	22	3%	0	100%	0	100%
Bonding system						
GC	473		2	99.6%	3	99.4%
VL	199		9	95.5%	26	86.9%
Overall	672		11	98.4%	29	95.7%

Of the 672 veneers studied, 11 failures were observed, resulting in a 98.4% survival rate. The distribution of failures is shown in Table 2.

**TABLE 2:** Failure type, reasons for failure, and number of failed veneers.

Complication type	Reason of failure	Number of restorations	Percentage
Mechanical complication	Fracture/chipping of ceramics	5	0.75%
	Debonding	1	0.15%
	Tooth loss	1	0.15%
Esthetic complication	Esthetic failure	3	0.45%
Biological complication	Post‐operative sensitivity	1	0.15%
**Total**		11	1.65%

Based on the occurrence time, three types of failures have been recorded:

– 2 short‐term complications, due to an esthetic failure and a post‐operative sensitivity, respectively, occurring at 6 to 7 months after bonding.
– 3 medium‐term complications: debonding, fracture, and tooth loss, occurring between the 45th and 68th month of follow‐up. The debonding was related to the complexity of the rubber dam placement. The veneer was subsequently rebonded without encountering any further complications.
– 6 long‐term complications, occurring around the 10th year of follow‐up, including 4 chippings in a patient with recurrent onychophagia, and two veneers (in the same patient) that were no longer aesthetically pleasing after bleaching.

**FIGURE 5:**
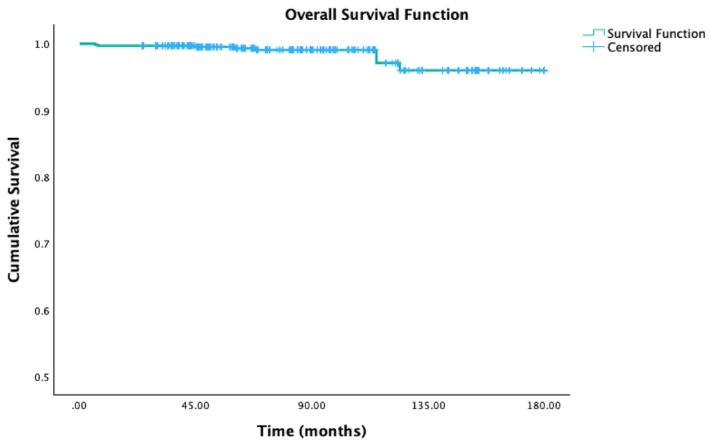
Overall time‐dependent survival estimator probability.

The overall survival probability (Figure 5) over the observation period of 180 months (15 years), mean observation period of 5.96 years was 96%, standard error of 0.13%.

Additionally, 18 minor complications, in 8 patients, have been recorded, without a need for veneer replacement. Seventeen of these complications were minor chippings, in a bruxism or onychophagia context, while the last minor complication was a veneer wear. One minor complication occurred 42 months after veneer bonding, while all the others occurred after more than 10 years of follow up.

**FIGURE 6:**
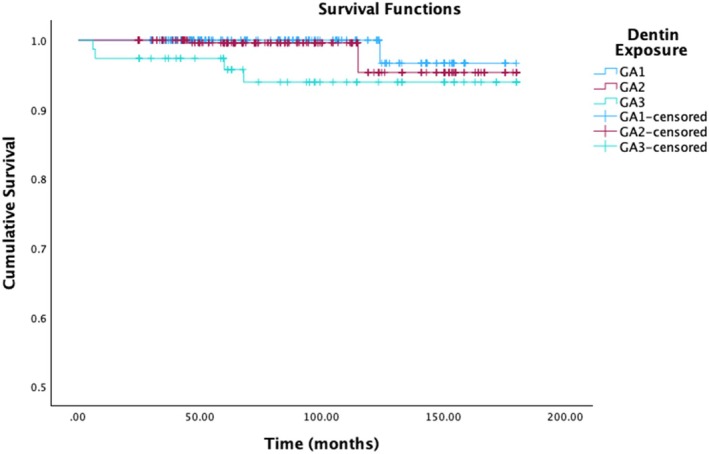
Overall time‐dependent survival estimator probability for dentin exposure groups. GA1 (enamel‐only), GA2 (< 30% dentin), and GA3 (> 30% dentin).

The estimated survival rate according to the degree of dentinal exposure is shown in Figure 6. For the 290 teeth where enamel was not exposed during preparation (GA1), the estimated survival rate was of 96.7% (standard error 2.3%) and 2 failures have been observed. These failures occurred after 10 years of follow‐up, for esthetic reasons, in the same patient. The group with up to 30% dentinal exposure during preparation (GA2) included 306 teeth, showing an estimated 95.3% survival rate (standard error 2.1%) with 5 failures. One of these failures occurred on a medium‐term basis, due to debonding. The other 4 failures occurred after almost 10 years of follow‐up, in a patient with recurrence of onychophagia. Finally, when the dentinal exposure was above 30% (GA3), a 93.9% survival rate was estimated (standard error 3%) with 4 failures. The reasons for these failures were variable: two early‐term failures occurred for biological or aesthetic reasons, respectively. The other two failures occurred after at least 5 years of follow‐up, due to a fractured tooth on a non‐vital tooth and a fracture on a vital tooth, respectively. These differences were statistically significant, *p =* 0.139, Kaplan–Meier, Log Rank (Mantel‐Cox). Pairwise comparison showed that the GA3 group was significantly different from the GA1 group, *p =* 0.033, Log Rank (Mantel‐Cox).

The odds ratio for failure, with any dentinal exposure, was 3.47. When the dentinal exposure was greater than 30% (GA3 group), the OR for failure was 4.67. The odds ratio for survival without any complications was 2.56 if any dentin was exposed.

**FIGURE 7:**
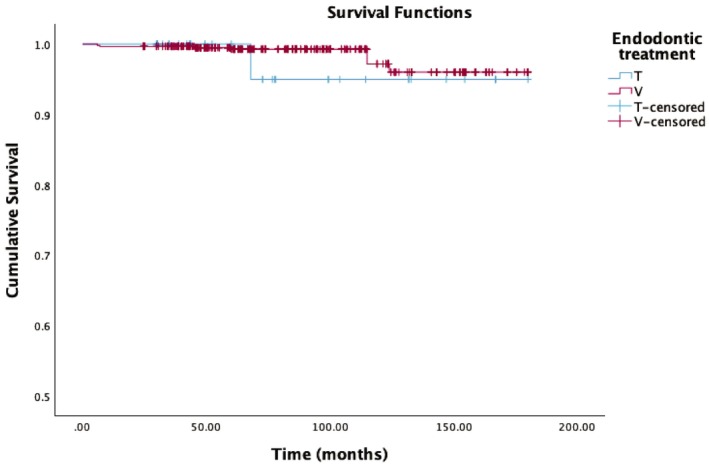
Overall time‐dependent survival estimator probability according to the presence or absence of endodontic treatment. (T: non‐vital teeth, V: vital teeth).

A survival analysis based on the pulpal status of the teeth was performed (Figure 7). The majority of teeth included in the study had not undergone endodontic treatment (*N* = 634), while 38 teeth were non‐vital. The estimated survival rate for the NVT group was 95.0% (standard error 4.9%) with one medium‐term failure observed on a GAC3 tooth. The VT group of 634 teeth showed a survival rate of 96.0% (standard error 1.4%) with 10 failures. No statistically significant difference was found between the survival rates of the two subgroups (*p* > 0.05).

For non‐vital teeth, the odds ratio for failure was 1.68. The OR for failure including minor complications was 1.24.

**FIGURE 8:**
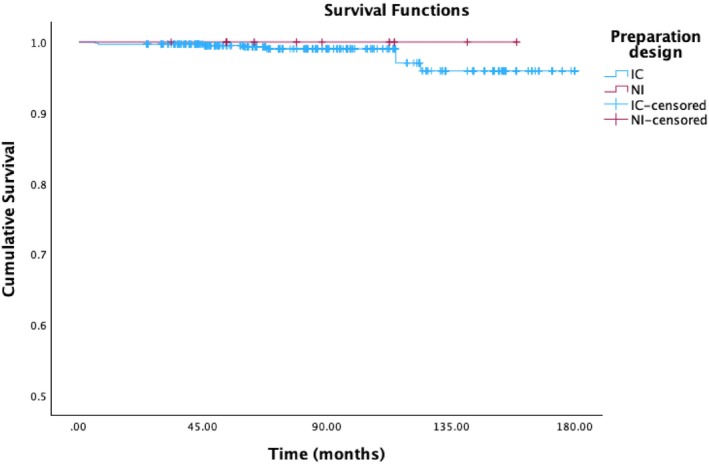
Overall time‐dependent survival estimator probability according to the preparation design. (IC = incisal coverage; NI = no incisal coverage).

Based on preparation design, there was no significant difference between the survival rates of the 2 groups, *p* > 0.05 (Figure 8). The number of teeth prepared with a butt margin design was 650 and there were 11 failures, for an estimated survival rate of 95.9%, standard error 1.3%. Of the 22 teeth with an incisal overlap design, no failures have been recorded.

**FIGURE 9:**
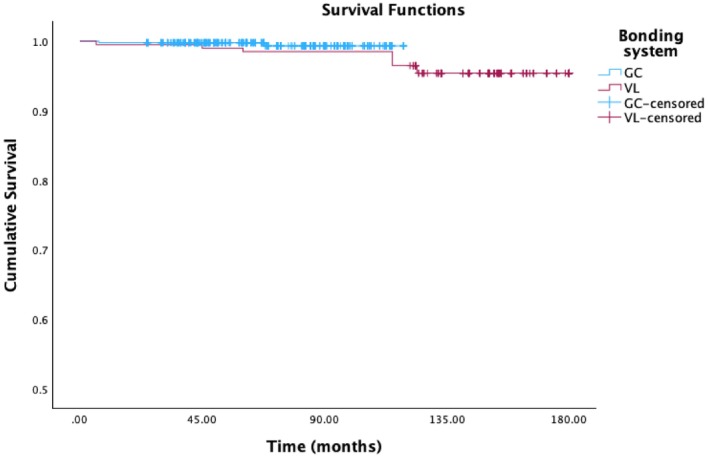
Overall time‐dependent survival estimator probability according to the bonding system used. (VL = Variolink system, GC = GC system).

Between 2001 and 2015, 199 restorations were bonded using the VL system, and from 2015 to 2023, the GC system was employed for the remaining 473 restorations. For the VL group, a 95.4% (standard error 1.5%) survival rate was estimated, with 9 failures. The failures that ensued are enumerated below: one early failure for esthetic reasons on a vital GA3 tooth, two medium‐term failures on vital teeth, with one debonding on a GA2 preparation, and one tooth fracture on a GA3 preparation; and lastly, 6 long‐term failures on vital teeth, with 4 chippings on GA2 teeth and 2 esthetic failures after bleaching. The GC group demonstrated a 99.3% estimated survival rate, standard error 0.5%, with 2 complications. One failure occurred in the shortterm for esthetic reasons on a GAC3 category vital tooth, while the other was a medium‐term complication due to a fractured non‐vital GA3 tooth. A comparison of the survival rates between the two subgroups (GC and VL) revealed no statistically significant difference (Figure 9).

## 4 Discussion

Ceramic veneers are widely recognized for their esthetic and functional benefits, with numerous studies highlighting their impressive clinical performance [1, 2, 3, 4, 5, 6, 7, 8, 9, 10]. Their survival rate without complications is reported as influenced by various factors, including the degree of dentin exposure, endodontic treatment, preparation design, and bonding system [1, 2, 3, 4, 5, 6, 7, 8, 9, 10].

This study primarily aimed to evaluate whether dentin exposure affects the survival rates of ceramic veneers, particularly when exceeding specific thresholds. Secondary objectives included examining the impact of endodontic treatment, preparation design, and resin cements on survival outcomes, and calculating the OR associated with dentinal exposure and endodontic treatment.

This retrospective study, with a follow‐up period up to 15 years and a mean follow‐up period of 5.98 years, demonstrated a highly favorable observed survival rate of 98.4% for ceramic veneers, comparable to other long‐term studies [3, 6, 25, 31, 38, 41]. Furthermore, the results of this research indicate an overall survival probability of 96%, a result consistent with other medium‐ and long‐term clinical studies [3, 6, 25, 31, 38, 41]. While veneers bonded exclusively to enamel did not achieve a 100% survival rate, they exhibited excellent performance, with failures primarily attributed to esthetic complications or poor patient habits rather than bonding defects. Conversely, veneers with dentin exposure showed an increased failure rate, corroborating prior studies [6, 25, 31, 41]. Therefore, the first research hypothesis was rejected. This study reinforces the importance of enamel preservation during preparation, as it significantly enhances the longevity and reliability of CLV.

Most teeth exhibited a moderate dentin exposure under 30% (GA2), commonly observed in the cervical third of incisors due to the minimal enamel thickness in this region (0.3–0.4 mm) and the required reduction of 0.3–0.5 mm [15, 16, 17]. The literature shows inconsistent findings regarding the impact of dentin exposure: some authors conclude that it was not possible to perform a meta‐analysis of the influence of enamel/dentin preparation on failure rates [9], while others [2, 37, 41, 42] highlight an increased failure risk when veneers are bonded to extensive areas of exposed dentin. A recent CBCT‐based (Cone Beam Computed Tomography) study has accurately mapped enamel thickness, reporting average cervical enamel thicknesses of 0.3–0.5 mm in anterior teeth, corroborating the risk of unintentional dentin exposure in these zones [16]. Hence, clinically, the use of depth‐gauge rotary instruments, mock‐up guided techniques, and preoperative wax‐ups, as employed in this study, is essential for guided and conservative preparations [1, 18, 43, 44].

The analysis revealed a significantly higher failure rate when veneers were bonded to substrates with more than 30% dentin exposure, aligning with previous findings [14, 22], though this study included a larger patient sample. In addition, in this study, an OR of 3.47 for failure was observed for veneers bonded on a stump with any dentinal exposure versus enamel only. When comparing increased dentinal exposure (GA3) to no (GA1) or minimal dentinal exposure (GA2), the OR was 4.67. Biomechanical considerations provide insights into these results [12, 13, 14, 25]. From a biomechanical perspective, dentin's lower modulus of elasticity and its organic‐rich composition contribute to it being a weaker adhesive substrate compared to enamel [12, 13, 14, 25]. The reduced stiffness of dentin, coupled with the presence of water and collagen, can significantly diminish the bond strength between dentin and restorative materials, increasing the risk of bond failure over time. This can also lead to increased microleakage, which is often associated with the ingress of bacteria and fluid that can lead to post‐operative sensitivity or caries. Furthermore, the increased susceptibility of dentin to mechanical stresses makes it more prone to delamination and other forms of failure, especially when subjected to masticatory forces. To circumvent such issues, some authors recommended that the IDS protocol be implemented [10]. However, using a filled adhesive (e.g., Optibond FL, Kerr), as usually recommended in this approach, would inevitably result in a thick adhesive layer over the exposed dentin, reaching a mean thickness of 87.99 + −77 μm [45]. Therefore, preliminary over‐milling would be necessary to accommodate this adhesive layer; otherwise, the precise fitting of the veneer and the marginal gap could be adversely affected, as it has been described [46].

Another point that has been addressed in favor of IDS is the better adhesive and mechanical behavior of CLV when bonded with an IDS protocol compared to a Delayed Dentin Sealing (DDS) protocol [47]. However, in the cited study [47], DDS was conducted without photopolymerization of the adhesive layer, contrary to the protocol followed in the present study. According to a recent publication, the outcome of a DDS with or without polymerization of the adhesive layer has a significant clinical impact [48]. This clinical point may explain why, even though the IDS protocol was not used to cover the exposed dentin in this study, only one case of veneer debonding was recorded, with no delamination or fracture observed.

Indeed, the failure rates observed were 1.7% and 4.3% for survival when minor complications were considered, with a total of 29 complications but only 11 failures over the 15‐year observation period. These rates are regarded as low, with the majority of complications being attributed to patient‐related factors.

In this study, all CLV were made of lithium disilicate ceramic. A recent meta‐analysis comparing feldspathic, leucite‐reinforced, and lithium disilicate veneers reported 10‐year survival rates of 96.1%, 93.7%, and 96.8%, respectively [38], in agreement with the findings in the present study. The most common complications observed in this retrospective study were ceramic fractures and chipping, consistent with other studies identifying fracture as the primary cause of failure [31, 49]. Although patients were advised about the risks associated with poor habits such as onychophagy, they were significant contributing factors, as reported by others [37, 50]. Furthermore, in this study, bruxism, widely recognized as a major risk factor [9, 37, 51], accounted for several failures, particularly minor chippings. To prevent sleep bruxism, the rigid occlusal splint worn during nocturnal sleep has been shown to be an effective method. However, it should be noted that many bruxism patients also suffer from awake bruxism, for which protection is not as easily obtained. Thus, patient selection and regular recalls can be considered key factors for CLV success over time.

Aesthetic complications included 3 cases requiring restoration replacement. Reasons included underlying discoloration of a non‐vital tooth and color mismatches due to subsequent tooth whitening conducted outside of the dental office. Thus, effective communication with patients about a possible future bleaching treatment can anticipate this type of complication.

Biological complications were rare, yet occurred in the short‐term, with only one case of post‐operative sensitivity requiring restoration replacement. A thorough examination of the photograph revealed dentin exposure in this clinical case (GA3 group). Consequently, the suspected cause was attributed to an elevated temperature during the preparation process, as reported in previous research [52].

Endodontically treated teeth exhibited a slightly lower survival rate (95%) compared to vital teeth (96%), but this difference did not reach statistical significance (*p* = 0.289) and is consistent with previous studies [23, 30]. Hence, the second research hypothesis was accepted. However, the lack of statistical significance may be attributed to the relatively small sample size of NVT (*N* = 38), limiting robust comparisons. In previous research, no significant difference was found in the survival between vital and non‐vital teeth over a 7‐year period, provided that proper adhesive protocols were used [23]. On the other hand, the calculated odds ratio for veneer failure on an NVT was 1.68. In the same way, this OR should be interpreted carefully as the observed failure rate for NVT was very low. The mechanical properties of endodontically treated teeth, such as reduced toughness and increased flexural strain, may partially explain the increased risk [31]. On the other hand, the survival of an endodontically treated tooth has been reported to be linked to the amount of remaining tooth substance [53] rather than the indirect restoration type [30, 53]. Thus, these findings do not necessarily contraindicate the use of CLVs on non‐vital teeth, provided that proper case selection and bonding techniques are applied.

No statistically significant difference in survival was observed between veneers with incisal coverage and those with a butt‐joint finish (*p* = 0.214). However, the limited number of incisal overlap designs (*n* = 21) makes the conclusion tenuous. It is noteworthy that the literature does not strongly support one preparation design over another in terms of survival [28]. Rather, substrate preservation, margin integrity, and avoidance of sharp internal angles remain the most important factors influencing performance. On the other hand, incisal overlap preparations, often used when masking incisal wear or discoloration, may inadvertently increase the risk of marginal stress concentration if improperly executed. Nevertheless, in this study, the controlled use of incisal reduction—always guided by diagnostic wax‐ups and confined to enamel when possible—appears to have reduced this risk. This supports prior findings that preparation design alone is not a primary determinant of veneer longevity, provided adhesive integrity is maintained [27, 28].

Cementation is a critical step in the veneer protocol. Yet, the choice of the bonding system and protocol is often practitioner dependent. Light‐cured resin cements are often used for thin, translucent veneers due to their color stability and extended working time [22]. At the same time, improvements in the composition of the dual‐cured cements have contributed to several clinical studies reporting favorable long‐term outcomes [6, 25, 36, 54]. Furthermore, in vitro studies comparing light‐cure and dual‐cure systems have shown comparable results [13]. In addition, in early 2001, no specific clinical guidelines were available regarding the polymerization mode of the resin cements for CLVs. Thus, the present study objectively reports the procedures, as they were conducted.

In this study, two adhesive systems and resin cements—VL and GC—were used, reflecting a change in the practitioner's protocol over time. The etch‐and‐rinse adhesives were associated with dual‐cure resin cements, chosen for their high fillers content. No significant difference in success rates was observed between these subgroups, and the fourth research hypothesis was accepted.

Yet, 3 failures due to esthetic complications have occurred in the VL group, aligning with literature citing the limitations of older dual‐cure systems containing aromatic amines, which are prone to yellowing and hydrolytic degradation [32, 33]. However, these results are challenging to interpret, as veneers bonded with the VL protocol were placed earlier, increasing the likelihood of failure due to longer exposure to wear and environmental factors. Moreover, scientific data suggest that VL is less prone to color changes, compared to other dual‐cured cements [33]. The findings regarding the GC group, which exhibited fewer failures, are supported by other research, that demonstrated that amine‐free dual‐cure cements achieve comparable color stability to traditional light‐cure cements [32], making them ideal for CLVs where esthetics and marginal integrity are critical. Additionally, the practitioner's growing experience, alongside advancements in preparation and bonding techniques emphasizing minimally invasive dentistry, may have contributed to the observed differences as demonstrated in vitro [20]. Furthermore, the overall survival rate observed in this study is comparable to studies having used light cured resin cements [10, 55]. These factors highlight the complex interplay between material selection, clinical protocols, and practitioner expertise in influencing the clinical outcomes.

Certain limitations inherent to this study merit attention. Firstly, longitudinal and retrospective studies are subject to an attrition bias during the observation phase. Furthermore, while the originality of this study lies in its extensive library of treatment photographs, these images are 2D and inherently limited in their ability to be standardized and to fully capture clinical details. Specifically, the palatal aspect of the preparations was not evaluated, leading to a partial loss of information. However, only a small number of teeth included such an incisal overlap design, and these were predominantly within enamel.

Secondly, photographs were analyzed using ImageJ software, with a free‐hand selection of the dentin and overall dental surface, which introduces potential inaccuracies. Differentiating between enamel and dentin in preparation images is sometimes challenging, as inter‐ and intra‐individual variability in hard tissue characteristics can reduce reproducibility. To mitigate this, exposure ranges were employed instead of precise values for defining sample groups. Nonetheless, for subgroup GA1 (0% dentin exposure), identifying the absence of dentin exposure was straightforward, and minor measurement errors had minimal impact on subgroup classification.

Lastly, in this retrospective study, survival rates were assessed using straightforward clinical criteria such as debonding, tooth fracture, or ceramic fracture needing a novel veneer. A robust analysis with an evaluation of the success rates would have been interesting, especially using standardized evaluation frameworks, such as the USPHS criteria, and systematic recall for clinical reassessment. This approach could have led to additional data regarding the color stability of the veneers alongside their respective resin cement.

Additionally, this study is based on the evaluation of CLV performed by a single operator, which offers the advantage of protocol, material, and data collection homogeneity, thereby enhancing the internal validity. However, this single‐centered nature of the study may be considered a limitation, due to its reduced external validity. While a multicentered study would have improved the reliability of the findings by increasing the sample size, it may also have introduced variability in protocols and data collection, potentially affecting the consistency of the results. Future research should consider these refinements to enhance the validity and applicability of study outcomes.

## 5 Conclusions

In conclusion, the results of this retrospective study, with a follow‐up ranging from 1 to 15 years and a mean follow‐up of 5.98 years, indicate that CLV demonstrates high survival rates, with an observed survival of 98.4% for 672 veneers. The observed success rate for veneers bonded exclusively to enamel was 99.3%, highlighting the critical role of enamel preservation during the preparation process. A significant increase in failure rates was observed when dentin exposure exceeded 30%. Endodontic treatment, preparation design, and the type of the dual‐cure bonding system did not significantly affect the survival of the CLVs.

## Conflicts of Interest

The authors declare no conflicts of interest.

## Data Availability

The data that support the findings of this study are available on request from the corresponding author. The data are not publicly available due to privacy or ethical restrictions.
